# Effect of sustained measurable residue disease negativity and post‐remission treatment selection on the prognosis of acute lymphoblastic leukemia in adults

**DOI:** 10.1002/cam4.7310

**Published:** 2024-05-24

**Authors:** Jiechen Yu, Yanrong Luo, Libing Wang, Tao Wang, Mingyu Ye, Jie Chen, Xiong Ni, Li Chen, Lei Gao, Jianmin Yang

**Affiliations:** ^1^ Department of Hematology Institute of Hematology, Shanghai Changhai Hospital, Naval Medical University Shanghai China

**Keywords:** acute lymphoblastic leukemia, allogeneic hematopoietic stem cell transplantation, measurable residual disease, prognosis

## Abstract

**Background:**

To explore the effects of monitoring measurable residual disease and post‐remission treatment selection on the clinical outcomes of B‐cell acute lymphoblastic leukemia (B‐ALL) in adults.

**Methods:**

Between September 2010 and January 2022, adult patients with B‐ALL who received combination chemotherapy, with or without allogeneic hematopoietic stem cell transplantation (allo‐HSCT), were included in the retrospective study, which was approved by the Ethics Committee and the observation of Declaration of Helsinki conditions.

**Results:**

One hundred and forty‐three B‐ALL patients achieved complete remission (CR) were included in the study, of whom 94 patients (65.7%) received allo‐HSCT in first complete remission (CR1).

Multivariate analysis showed that the most powerful factors affecting OS were transplantation (hazard ratio [HR] = 0.540, *p* = 0.037) and sustained measurable residue disease (MRD) negativity (HR = 0.508, *p* = 0.037). The subgroup analysis showed that the prognosis of the allo‐HSCT group was better than that of the chemotherapy group, regardless of whether MRD was negative or positive after two courses of consolidation therapy. After consolidation therapy, the prognosis of patients with positive MRD remained significantly better in the allo‐HSCT group than in the chemotherapy group. However, no significant difference was observed in the prognosis between the allo‐HSCT and chemotherapy groups with negative MRD after consolidation therapy.

**Conclusions:**

B‐ALL patients who achieve sustained MRD negativity during consolidation therapy have excellent long‐term outcomes even without allo‐HSCT. Allo‐HSCT is associated with a significant benefit in terms of OS and DFS for patients who were with positive MRD during consolidation therapy.

## 1 INTRODUCTION

Acute lymphoblastic leukemia (ALL) is a hematologic malignancy that accounts for approximately 20% of all cases of adult leukemias.^1^ With the use of pediatric‐inspired protocols, survival rates for adult patients with ALL has significantly increased over the last decade.^2^ However, less than 40% of adult ALL patients achieve long‐term survival, while older patients aged 55 or more years still have a dismal prognosis with overall cure rates of less than 20%.^3^ Nowadays, consolidation with allo‐HSCT is recommended for adult ALL patients in first complete remission (CR1) but with persistent measurable residue disease (MRD), or negative MRD but with baseline high‐risk features, although the definitions of high risk are highly heterogenous.^4^, ^5^ Previous meta‐analyses of published randomized trials on post‐remission therapy (PRT) in ALL adults reported a significant reduction in all‐cause mortality with allo‐HSCT in CR1 compared with chemotherapy.^4^, ^6^, ^7^ Reciprocally, our systematic review demonstrated that pediatric‐inspired chemotherapy conferred a superiority over allo‐HSCT for adult ALL in CR1.^8^ Therefore, increasing attention has been paid to the question whether allo‐HSCT is still a necessary component of therapy for all ALL patients.

Monitoring measurable residual disease (MRD) has been increasingly used in patients with ALL, and emerged as a powerful stratification tool, which can identify patients more likely to benefit from allo‐HSCT in first CR.^9^ Several studies have proved that MRD negative, assessed by flow cytometry, have been associated with improved outcomes, both pre and posttransplantation.^10^, ^11^, ^12^ Another interesting issue is, should anyone with ALL who obtains sustained undetectable MRD receive a transplant in first remission, since non‐relapse mortality (NRM) after allo‐HSCT remains an unsolved clinical problem.

In this study, we retrospectively analyzed the predictive value of MRD assessment by flow cytometry in adult ALL patients receiving combination chemotherapy, with or without allo‐HSCT. We also evaluated the impact of allo‐HSCT on survival of patients with sustained undetectable MRD.

## 2 METHODS

### 2.1 Patients

Between September 2010 and January 2022, 143 ALL patients (86 male, 72 female) treated in Changhai hospital were included in the study, excluding chronic myeloid leukemia acute lymphoblastic transformation. Ninety‐nine patients were initially assessed and treated at Changhai hospital, and 44 patients were referred for further treatment after initial therapy elsewhere. CR was achieved after 1–2 cycles of induction, and all patients had MRD assessment by RT‐qPCR at both CR and thereafter. The median age of the 143 patients at diagnosis was 35 years (range; 16–76 years). Central nervous system involvement at presentation was identified in 3.5% (5/143) of patients. Institutional databases were retrospectively reviewed; demographic, clinical and genetic information were extracted from the medical records. The study protocol was approved by the Ethics Committee of Changhai hospital. The requirement for written informed consent was waived off, because this study used retrospective data from medical records, and there were no interventions in the patients. ALL diagnosis was characterized according to the WHO classification schemes.

### 2.2 Chemotherapy

Two chemotherapy backbone regimens were used: Hyper‐CVAD/MA based and CH‐ALL2013 based. The details of chemotherapy regimens see details in Table S1. Forty two patients received Hyper‐CVAD/MA regimen, whereas 101 patients were treated with CH‐ALL2013 based chemotherapy. Patients who did not reach hematologic CR after 2 cycles of induction were not included in the study. Prophylaxis of central nervous system leukemia consisted of intrathecal chemotherapy with methotrexate, cytarabine, and dexamethasone for at least six doses during induction and consolidation chemotherapy. All patients enrolled onto the study received a minimum of two blocks of consolidation chemotherapy.

### 2.3 Transplant procedures

Allo‐HSCT was performed in 94/143 (65.7%) CR1 patients. The procedure was all myeloablative. Sixty‐nine patients (69/94, 73.4%) received transplant from HLA‐matched related or unrelated donor, and 25 patients (26.6%) received haplo‐matched transplantation. The conditioning regimen was as reported.^13^, ^14^ Graft versus host disease (GVHD) prophylaxis regimen consisted of cyclosporine A (CsA), mycophenolate mofetil (MMF), and short‐term methotrexate.^13^

### 2.4 MRD definitions and detection

MPFC was performed with a panel of antibodies designed for B‐ALL. The diagnostic panel for B‐ALL contained CD45, CD7, CD19, CD13, CD33, CD34, CD117, HLA‐DR, CD10, cMPO, cCD3 and cCD79a and its extended panel included CD66c, CD22, CD20, CD58, CD38, CD123, CD45, cytoplasmic heavy chain of immunoglobulin M(cu) and cytoplasmic TDT (BD Bioscience; San Jose, CA; Beckman‐Counter; Indianapolis, IN).^15^ FACS Aria II (BD Biosciences) with Diva program was used to acquire and analyze the data. MRD panel included CD58‐FITC, KORSA‐PE, CD34‐Percp, CD20‐PE‐cy7, CD10‐APC, CD19‐APC‐H7, CD38‐V450 and CD45‐V500 (BD Bioscience and Beckman‐Counter).

Negative MRD by MPFC was defined as <10^−4^ blasts (0.01%) in bone marrow samples and Flow‐MRD positivity was defined as >10^−4^ blasts (0.01%) in bone marrow. In our study, patients with sustained undetectable MRD were defined according to negative MRD results observed at the end of consolidation and any of the previous time points.

### 2.5 Statistics

Pair‐wise comparisons between patients' characteristics (covariates) were performed using the Mann–Whitney test or Kruskal‐Wallis test for continuous variables and with the Fisher's exact test for categorical variables. Patients alive in CR were censored at the time of last contact. OS and DFS were estimated by the Kaplan–Meier method and compared using the log‐rank test. The CIR was compared between groups using the method of Gray, with estimation determined by the method of Kalbfleisch and Prentice.^16^ Hazard ratios are given with 95% confidence intervals (95% CI). Survival‐time data (DFS and OS) and covariates were analyzed using the method of Cox proportional hazards regression. All calculations were performed using the SAS 9.2 software package.

## 3 RESULTS

### 3.1 Patient characteristics

The whole cohort comprised 143 evaluable patients with B‐ALL in CR1. At diagnosis, the median leukocyte count was 12.03 × 10^9^/L (range; 0.55–489 × 10^9^/L). According to the karyotype analysis and molecular alterations, patients were classified into three subgroups: Ph^+^ ALL (*n* = 57, 39.9%), Standard risk Ph^−^ ALL (*n* = 27, 18.9%), and High risk Ph^−^ ALL (*n* = 59, 41.2%). The patients' characteristics are summarized in Table 1.

**TABLE 1:** Basic characteristics of patients.

Characteristic	Total (*n* = 143)	Chemo (*n* = 49)	HSCT (*n* = 94)	*p* value
Male [*n* (%)]	69 (48.3)	19 (38.8)	50 (53.2)	0.102
Age [years of age, median (range)]	35 (16 ~ 76)	49 (17 ~ 76)	30 (16 ~ 61)	<0.001**
Presenting WBC [×10^9^/L, median (range)]	12.03 (0.55 ~ 489)	9.82 (0.81 ~ 309)	14.95 (0.55 ~ 489)	0.797
4‐year OS (95% CI)	76.7% (73.1%–80.3%)	42.1% (33.2%–51%)	73.9% (69.2%–78.6%)	0.039*
4‐year DFS (95% CI)	72.2% (68.4%–76%)	40.4% (32.3%–48.5%)	69.2% (64.3%–74.1%)	0.021*
4‐year CIR (95% CI)	23.5% (23.1%–23.9%)	28% (27.5%–28.5%)	17.9% (17.45%–18.35%)	0.196

*Note*: *p < 0.05; **p < 0.001.

Abbreviations: CI, confidence interval; CIR, cumulative incidence of relapse; DFS, disease‐free survival; HSCT, allogeneic hematopoietic stem cell transplantation; OS, overall survival; WBC, white blood cell count.

### 3.2 Overall outcomes

The median follow‐up for surviving patients treated between September 2010 and January 2022 were 54.8 months (range; 3.4–153.9 months). The last follow‐up was January 1, 2023. For the cohort of 143 patients, the 5‐year CIR was 22% (95% CI: 18.1%–25.9%), DFS was 67.5% (95% CI: 63.5%–71.5%), and OS was 73.4% (95% CI: 69.6%–77.2%). The overall relapse rate was 19.6% (28/143) in this cohort.

**FIGURE 1:**
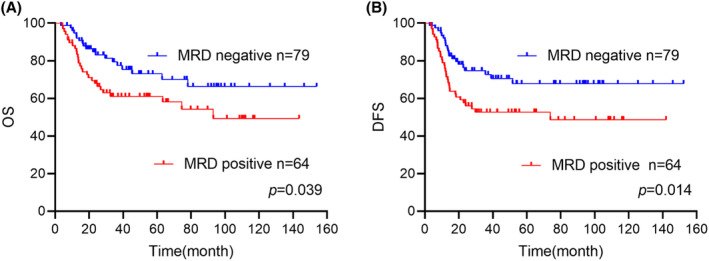
(A, B) Comparison of overall survival (OS), disease‐free survival (DFS) between measurable residue disease (MRD) negative and positive patients before consolidation therapy.

Seventy‐nine (55.2%) patients achieved MRD negative before consolidation therapy, while persistent MRD was detected in rest 64 (44.8%) patient samples. Patients who achieved MRD negative had a significantly superior OS as compared to those who did not achieve (MRD negative: 66.3% [95% CI: 59.3%–73.3%] vs. MRD positive: 49.3% [95% CI: 41.3%–57.3%], *p* = 0.039 Figure 1A). The same trend was observed for DFS. (Figure 1B).

**FIGURE 2:**
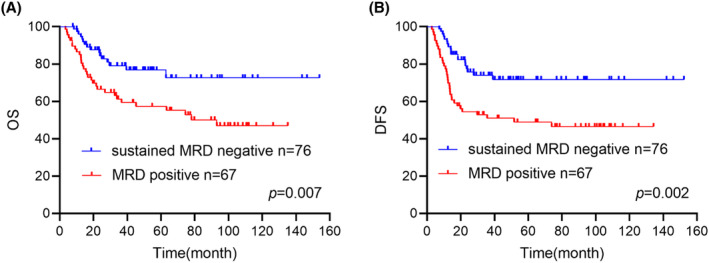
(A, B) Comparison of OS and DFS between patients with sustained MRD negative or not.

After completing consolidation therapy, ninety‐seven (67.8%) patients achieved MRD negative, of whom 76 patients achieved sustained minimal residual disease negativity. Sustained MRD negative was associated with a significant reduction in the risk of relapse and/or death at the end of consolidation therapy. (sustained MRD negative: 72.6% [95% CI: 66.1%–79.1%] vs. MRD positive: 47% [95% CI: 40%–54%], *p* = 0.007 Figure 2A). The same trend was observed for DFS. (Figure 2B).

### 3.3 Allo‐HSCT

All patients with CR had the option to proceed to allo‐HSCT. Ninety‐four of 143 (65.7%) patients treated with alloHSCT from sibling (*n* = 30), unrelated donor (*n* = 39), and haploidentical donors (*n* = 25). In the remaining 49 patients, the reasons for not undergoing alloHSCT included advanced age (*n* = 7), lack of a suitable donor (*n* = 15), early death (*n* = 2), poor performance status (*n* = 3), early relapse (*n* = 2), or patient refusal (*n* = 20).

Of 25 deaths after allo‐HSCT, the most common cause was relapse (44%), infection (20%), organ failure (24%), and GvHD (12%) were major four causes of NRM.

**FIGURE 3:**
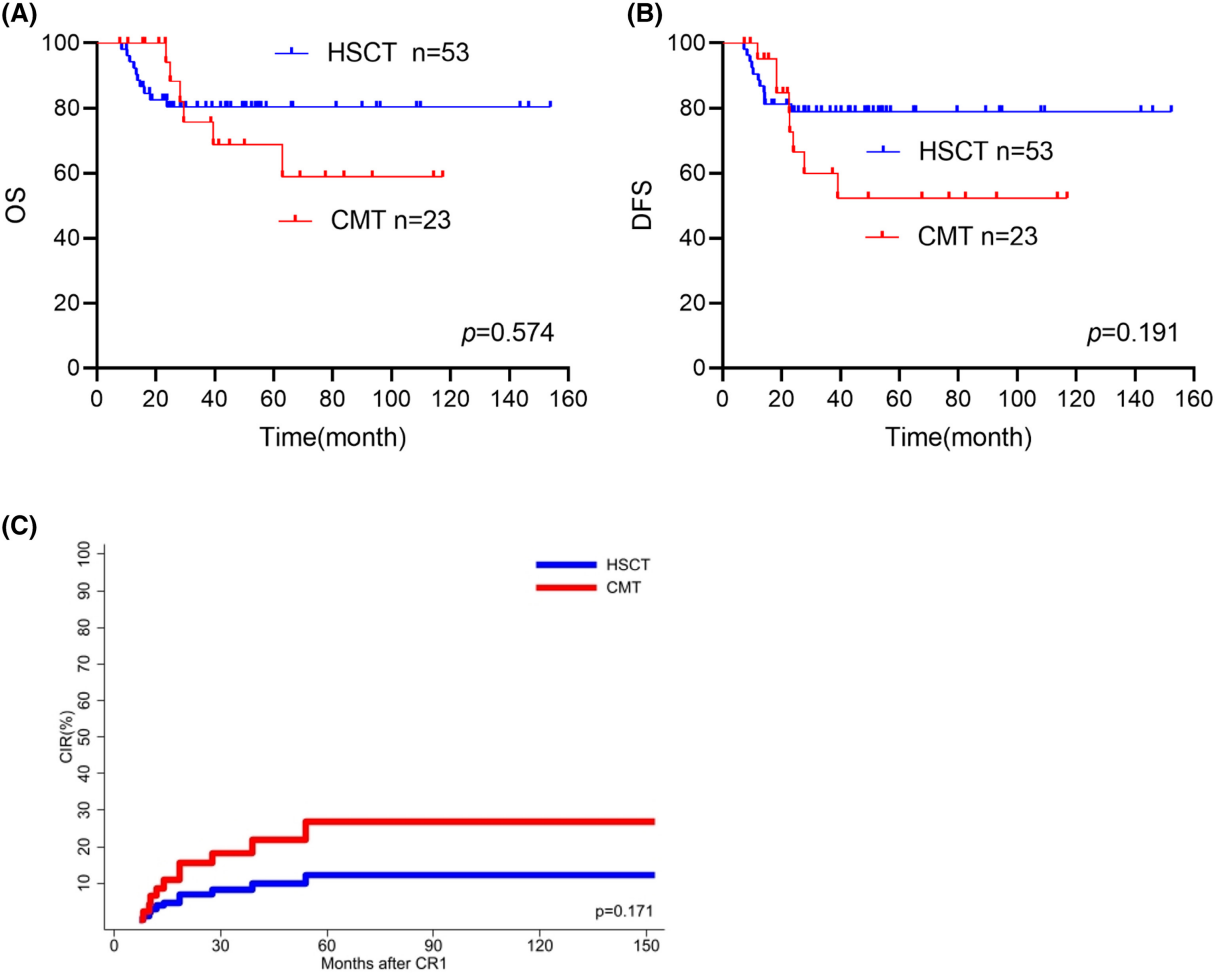
(A, B, C) Comparison of OS, DFS and cumulative incidence of relapse (CIR) according to different post‐remission treatment regimens in patients with sustained MRD negative.

No significant differences were observed in OS and DFS for patients with sustained undetectable MRD who received chemotherapy alone versus patients who treated with allo‐HSCT (OS: chemotherapy: 58.9%[95% CI: 45.4%–72.4] vs. allo‐HSCT: 80.4% [95% CI: 74.8%–86%], *p* = 0.574; DFS: chemotherapy: 52.4% [95% CI: 39.8%–65%] vs. 78.9% [95% CI: 73.2%–84.6%], *p* = 0.191; Figure 3A,B). The CIR rates was higher in chemotherapy group when compared with allo‐HSCT group. (27.6% [95% CI: 27.14%–28.05%] v,s, 12.5% [95% CI: 12.18%–12.81%], *p* = 0.171, Figure 3C).

**FIGURE 4:**
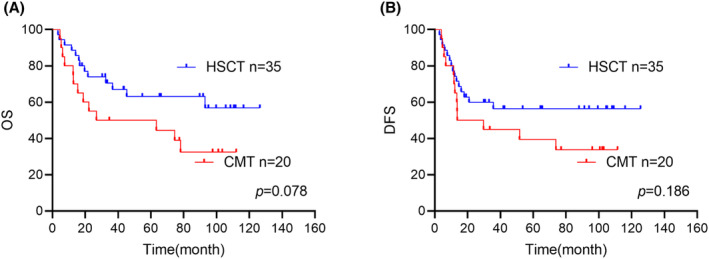
(A, B) Comparison of OS and DFS according to different post‐remission treatment regimens in patients with positive MRD after consolidating two regimens.

After two courses consolidation therapy, 55 patients did not achieve sustained undetectable MRD response, of whom 35 patients treated with allo‐HSCT as consolidation. We found a trend of the difference between the rates of survival in these two treatment modalities. (2‐year OS: chemotherapy: 32.4% [95% CI (31.3%, 33.5%)] vs. allo‐HSCT: 56.8% [95% CI (47%, 66.6%)] *p* = 0.078; 2‐year DFS: chemotherapy: 32.4% [95% CI: (31.3%, 33.5%)] vs. alloHSCT: 56.3% [95% CI (47.8%, 64.8%)], *p* = 0.186; Figure 4A,B). Relapse was the main factor affecting OS and DFS, with 2‐year CIR increasing from 10% [95% CI (6.2%, 13.8%)] in allo‐HSCT group to 18.1% [95% CI (16.4%, 19.8%)] in chemotherapy group. (*p* = 0.162).

### 3.4 Multivariate analysis

**FIGURE 5:**
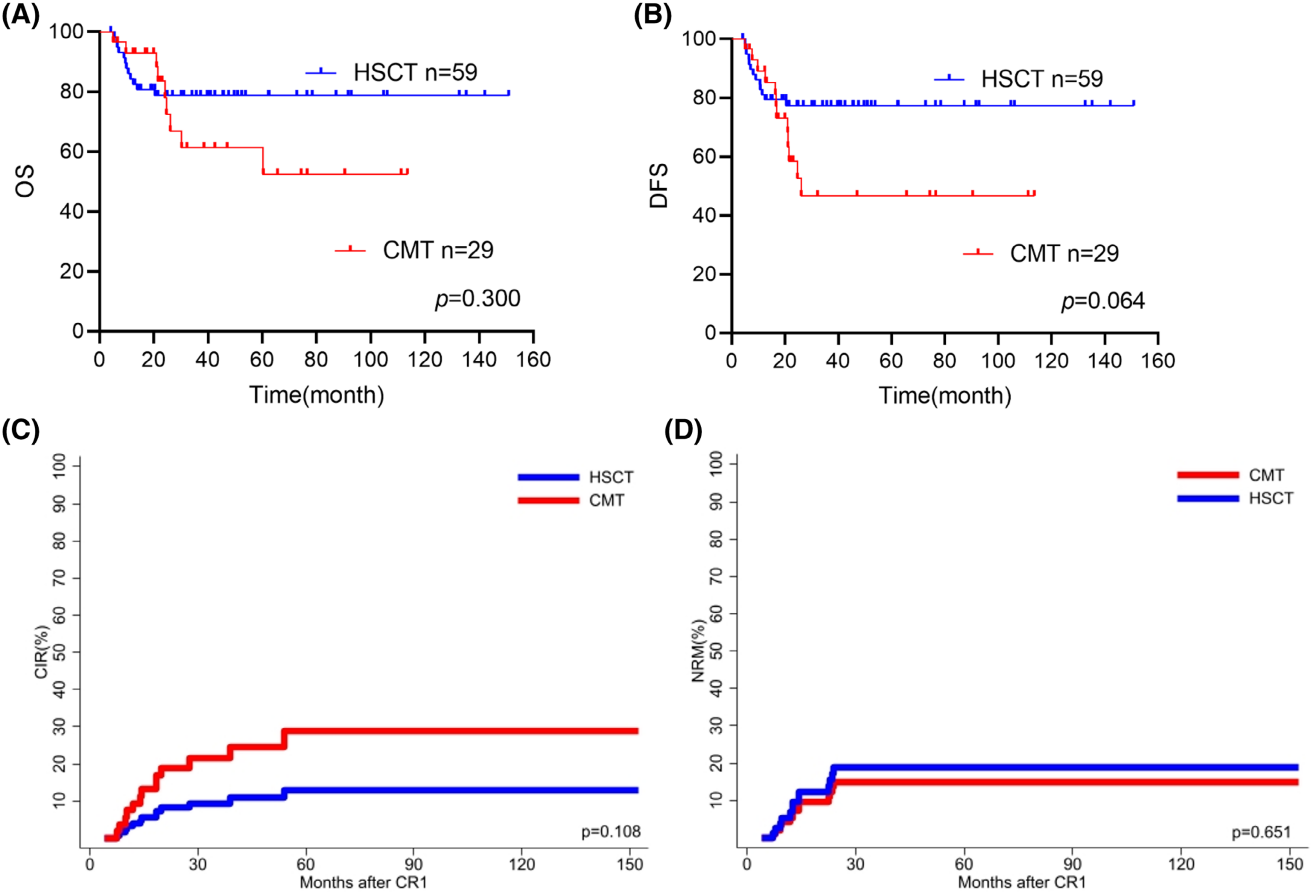
(A–D) Comparison of OS, DFS, CIR and non‐relapse mortality (NRM) according to different post‐remission treatment regimens in patients with negative measurable residue disease (MRD) after consolidating two regimens.

Eighty‐eight patients achieved sustained MRD negativity after two blocks of consolidation chemotherapy (88/143, 61.5%). The overall characteristics of the analyzable patients are shown in Table 2. Fifty‐nine patients (67%) received alloHSCT as consolidation, and 29 patients were treated with chemotherapy. Patients in the two groups were comparable for most variables. (Table 2) At last follow‐up, 15 (17%) patients had experienced relapse, whereas 13 (14.8%) patients died due to NRM. The 4‐year OS and DFS for patients with sustained MRD negativity after 2 blocks of consolidation chemotherapy were 73.5% (95% CI: 68.3%–78.7%) and 68.2% (95% CI: 62.8%–73.6%), respectively. For patients with sustained MRD negativity who treated with chemotherapy, 4‐year OS and DFS were 52.7% (95% CI: 40.2%–65.2%) and 46.4% (95% CI: 35.2%–57.6%), respectively. And for patients treated with alloHSCT, OS and DFS were 78.6% (95% CI: 73.1%–84.1%) and 77.3% (95% CI: 71.8%–82.8%), respectively. (Table 2, *p* values were nonsignificant for both comparisons, Figure 5A,B) There was a trend for higher CIR in chemotherapy group chemotherapy group: 28.5% [95% CI: 27.83%–9.17%] vs. alloHSCT group: 12.4% [95% CI: 11.95%–12.85%], *p* = 0.108; (Figure 5C) and for higher CI‐NRM in alloHSCT group. chemotherapy: 15.2% [95% CI:14.91–15.49] vs. 19.8% [95% CI:19.14%–20.46], *p* = 0.651; (Figure 5D).

**TABLE 2:** Basic characteristics of patients who achieved sustained MRD negativity after 2 blocks of consolidation chemotherapy.

Characteristic	Total (*n* = 88)	Chemo (*n* = 29)	HSCT (*n* = 59)	*p* value
Male [*n* (%)]	42 (47.7)	10 (34.5)	32 (54.2)	0.112
Age [years of age, median (range)]	35.5 (16 ~ 71)	49 (25 ~ 71)	30 (16 ~ 57)	<0.001**
Presenting WBC [×109 /L, median(range)]	11.585 (0.55 ~ 327)	11.55 (0.81 ~ 239.3)	11.62 (0.55 ~ 327)	0.938
4‐year OS(95% CI)	73.5% (68.3%–78.7%)	52.7% (40.2%–65.2%)	78.6% (73.1%–84.1%)	0.3
4‐year DFS(95% CI)	68.2% (62.8%–73.6%)	46.4% (35.2%–57.6%)	77.3% (71.8%–82.8%)	0.064
4‐year CIR(95% CI)	18.3% (17.63%–18.97%)	28.5% (27.83%–29.17%)	12.4% (11.95%‐12.85%)	0.108

*Note*: **p < 0.001.

Abbreviations: CI, confidence interval; CIR, cumulative incidence of relapse; DFS, disease‐free survival; HSCT, allogeneic hematopoietic stem cell transplantation; OS, overall survival; WBC, white blood cell count.

Univariate and multivariate analysis for DFS and OS is shown in Table 3. Allo‐HSCT and sustained MRD negative were both prognostic for OS and DFS. In multivariate analysis, Allo‐HSCT (*p* = 0.037; HR 0.54 [95% CI: 0.303–0.965])and sustained MRD negative (*p* = 0.037; HR 0.508 [95% CI: 0.27–0.95])were prognostic for OS, and sustained MRD negative (*p* = 0.018, HR 0.5 [95% CI: 0.28–0.89]), and treatment modalities (chemotherapy vs. alloHSCT; *p* = 0.024; HR 1.87 [95% CI: 1.08–3.23]) were prognostic for DFS.

**TABLE 3:** Single and multivariate analysis of clinical characteristics and prognosis of 143 patients with B‐cell acute lymphoblastic leukemia.

Prognostic factor	*n* (%)	Univariate survival analysis		Multivariate survival analysis					
				OS			DFS		
		*p*‐value (OS)	*p‐*value (DFS)	HR	95% CI	*p*‐value	HR	95% CI	*p*‐value
Sex		0.199	0.248						
Male	69 (48.3)								
Female	74 (51.7)								
Age at diagnosis		0.182	0.343						
<40 years	86 (60.1)								
≥40 years	57 (39.9)								
Presenting WBC (/10^9^/L)		0.272	0.338						
<30	104 (72.7)								
≥30	39 (27.3)								
Cytogenetic classification		0.096	0.109						
Ph^+^	57 (39.9)								
Ph^−^ with Adverse	27 (18.9)								
Ph^−^ with Neutral	59 (41.2)								
HSCT in CR1		0.039	0.021			0.037			0.024
No	49 (34.3)			1			1		
Yes	94 (65.7)			0.540	0.303,0.965		0.534	0.310,0.922	
MRD of consolidating two courses		0.028	0.011						
Negative	88 (61.5)								
Positive	55 (38.5)								
EOC‐MRD		0.131	0.019						
Negative	97 (67.8)								
Positive	46 (32.2)								
Sustained MRD negativity		0.007	0.002			0.037			0.018
Yes	76 (53.1)			1			1		
No	67 (46.9)			1.968	1.051,3.682		2.019	1.129,3.612	

Abbreviations: CI, confidence interval; DFS, disease‐free survival; EOC‐MRD, MRD at the end of consolidation; HSCT in CR1, allogeneic hematopoietic stem cell transplantation during first remission; MRD, measurable residue disease; OS, overall survival; WBC, white blood cell count.

## 4 DISCUSSION

The introduction of pediatric‐inspired chemotherapy regimen has revolutionized the management of ALL patients with several studies demonstrating improved outcome.^17^, ^18^ Modern multiagent therapy was effective in achieving a negative MRD, which may attribute to a better outcome. In this study, we demonstrated that achieving sustained MRD negativity was associated with a decreased likelihood of relapse and with a longer OS. Although MRD monitoring has been accepted as risk stratification tool, nevertheless, the optimal monitoring time point remains inconclusive.^19^, ^20^, ^21^

In a retrospective analysis of Ph^+^ ALL patients, there was a weak relationship between post‐induction MRD status and OS, RFS.^22^ While, pre‐HSCT MRD negativity, was associated with a decreased relapse rate in several studies.^22^, ^23^, ^24^, ^25^ In our small‐scale cohort, 76 patients got sustained MRD negativity. The OS was significantly improved in sustained MRD negative patients when compared with patients who had less MRD response. (72.6% vs. 47%, *p* = 0.007) Our data exhibited a good correlation of sustained MRD negativity with less relapse and better OS in B‐ALL patients. Among the 67 patients who did not reach sustained MRD negativity, 18 patients (26.9%) have relapsed. While, in 76 patients with sustained MRD negativity, 10 patients (13.2%) relapsed. The higher proportion of deep response contributed to improved DFS and OS rates. The CIR rate was higher in patients with chemotherapy when compared with patients who treated with alloHSCT. (28% in chemotherapy group and 17.9% in alloHSCT group, *p* = 0.196, Table 1). While, for patients who achieved sustained MRD negativity, the CIR was almost equivalent in both chemotherapy and alloHSCT groups.

AlloHSCT still remains the mainstay for adult ALL, but is compromised by NRM, so the evidence from studies successfully adopting multiagent therapy schedules has been considered.^9^, ^18^, ^26^, ^27^, ^28^, ^29^ The first group to suggest that alloHSCT could be omitted was Children's Oncology group. In 2009, Schultz et al. reported that imatinib combine with chemotherapy in pediatric patients between the ages of 1 and 21 showed that continuous imatinib exposure improved EFS at 3 years from the historical control of 35% up to 80% and suggested that EFS with chemotherapy plus imatinib was equal to or better than a sibling donor alloHSCT (88% vs. 57%, respectively).^18^ In a long‐term follow‐up report, the DFS at 5 years for chemotherapy plus imatinib was 70% compared to 65% for sibling donor alloHSCT and 59% for unrelated donor alloHSCT.^28^ We previous work showed that patients with complete molecular remission have almost equivalent survival with those received consolidation therapy as alloHSCT, but with slightly increased relapse rate.^30^ Although there was a trend for better survival among the patients treated with chemotherapy, this was not statistically significant. The higher NRM in patients underwent alloHSCT may contribute to discrepancy. For patients who were failed to acquire deep MRD response, alloHSCT should recommended over chemotherapy owing to superior outcome. For Ph^−^neg adult ALL, PETHEMA ALL‐HR‐11 trial demonstrated that sparing allo‐HSCT does not hamper the outcomes of patients up to 60 years of age.^31^ Our recent systematic review also did not observe the benefit of allo‐HSCT in HR patients. Neither did the subgroup analysis based on risk categories reveal significant differences in OS or DFS between the allo‐HSCT and chemotherapy arms for both the HR and SR groups.^32^ Nowadays, in the era of immunotherapy, several groups recently reported good outcomes in adult ALL patients treated with novel therapies such as monoclonal antibodies and CAR‐T cells.^33^, ^34^ Therefore, the optimal treatment of adult patients with B‐ALL in CR1 still remains to be established because the risk/benefit ratio associated with allo‐HSCT.

We acknowledge the limitations of our study, including the retrospective nature as well as the small sample size. Prospective trials using MRD‐based risk stratification for patient with B‐ALL may elucidate the optimal post‐remission management. In conclusion, acquisition of sustained MRD negativity was a strong prognostic factor in B‐ALL patients, even for patients treated with chemotherapy. For patients did not achieve MRD negative or loss MRD negativity during chemotherapy, alloHSCT was a potent strategy for better OS and DFS when compared with chemotherapy.

## AUTHOR CONTRIBUTIONS

**Jiechen Yu:** Data curation (lead); formal analysis (lead); writing – original draft (lead). **Yanrong Luo:** Data curation (equal). **Libing Wang:** Methodology (supporting); project administration (supporting). **Tao Wang:** Data curation (supporting). **Mingyu Ye:** Data curation (supporting). **Jie Chen:** Project administration (supporting). **Xiong Ni:** Project administration (supporting). **Li Chen:** Project administration (supporting). **Lei Gao:** Project administration (supporting). **Jianmin Yang:** Methodology (lead); project administration (lead).

## FUNDING INFORMATION

This research received no funding.

## CONFLICT OF INTEREST STATEMENT

Authors have no conflict of interest to declare.

## ETHICS STATEMENT

This study was approved by the Medical Ethics Committee of Changhai Hospital, Naval Military Medical University.

## CONSENT

The Ethics Committee of the Changhai Hospital, Naval Military Medical University waived the need to obtain consent for the collection, analysis and publication of the retrospectively obtained and anonymized data for this non‐interventional study.

## Supporting information


Table S1.

## Data Availability

The datasets generated and/or analyzed during the current study are not publicly available to protect patient privacy, but are available from the corresponding author on reasonable request.
