# Supplementary material for: Effect of sustained measurable residue disease negativity and post‐remission treatment selection on the prognosis of acute lymphoblastic leukemia in adults
Source: Cancer Med. 2024 May 24;13(10):e7310. doi: 10.1002/cam4.7310 (PMC11117453; doi:10.1002/cam4.7310)
Supplement: Supplementary file 1 — Table S1. [file CAM4-13-e7310-s001.docx]

| **Table S1. CH-ALL-2013 regimen** | | | | |
| --- | --- | --- | --- | --- |
|  | **Drug** | **Dose** | **Method** | **Time** |
| **Induction** | vincristine | 1.4 mg/m^2^ (maximum, 2mg) | IV | D1, 8, 15, 22 |
|  | daunorubicin | 45 mg/m^2^ | IV | D1-3,15-17 (depend on bone marrow at D14) |
|  | dexamethasone | 10 mg/m^2^ | IV | D1-14 |
|  |  |  |  |  |
| **Block 1** | cytarabine | 2 g/m^2^/q12h | IV | D1-2 |
|  | dexamethasone | 8 mg/m^2^/12h | IV | D1-2 |
| **Block 2** | methotrexate | 3 g/m^2^ | IV (over 24 hours) | D1 |
|  |  | after 12 hours the MTX given, give luecoorin about 5-8% dosage of MTX to rescue |  |  |
|  | vincristine | 1.4 mg/m^2^ ( maximum, 2mg ) | IV | D1 |
|  | 6-mercaptopurine | 75 mg/m^2^ | Po, at bedtime | D1-7 |
|  | dexamethasone | 10 mg/m^2^ | IV | D1-5 |
| **Block 3** | etoposide | 165 mg/m^2^ | IV | D1-3 |
|  | cytarabine | 75 mg/m^2^ | IV | D1-7 |
| **Block 4** | vincristine | 1.4 mg/m^2^ (maximum, 2mg) | IV | D1, 8, 15, 22 |
|  | daunorubicin | 45 mg/m^2^ | IV | D1-3 |
|  | dexamethasone | 10 mg/m^2^ | IV | D1-14 |
| **Block 5** | cytarabine | 100 mg/m^2^ | IV | D1-7 |
|  | cyclophosphamide | 1 g/m^2^ | IV | D1 |
|  | 6-mercaptopurine | 75 mg/m^2^ | Po, at bedtime | D1-7 |
| **Block 6** | methotrexate | 3 g/m^2^ | IV (over 24 hours) | D1 |
|  |  | after 12 hours the MTX given, give luecoorin about 5-8% dosage of MTX to rescue |  |  |
|  | vincristine | 1.4 mg/m^2^ (maximum, 2mg) | IV | D1 |
|  | 6-mercaptopurine | 75 mg/m^2^ | Po, at bedtime | D1-7 |
| **Maintenance** | vincristine | 2 mg | IV | q m |
|  | prednisone | 100 mg | Po | D1-7/ month |
|  | 6-mercaptopurine | 75 mg/m^2^ | Po | D1-5 / month |
|  | methotrexate | 10 mg/m^2^ | IV (over 24 hours) | q w |
